# Formation of visual memories controlled by gamma power phase-locked to alpha oscillations

**DOI:** 10.1038/srep28092

**Published:** 2016-06-16

**Authors:** Hyojin Park, Dong Soo Lee, Eunjoo Kang, Hyejin Kang, Jarang Hahm, June Sic Kim, Chun Kee Chung, Haiteng Jiang, Joachim Gross, Ole Jensen

**Affiliations:** 1 Department of Nuclear Medicine, Seoul National University College of Medicine, Seoul, Korea; 2 Institute of Neuroscience and Psychology, University of Glasgow, Glasgow, United Kingdom; 3 Institute of Radiation Medicine, Medical Research Center, Seoul National University, Seoul, Korea; 4 Interdisciplinary Program in Cognitive Science, Seoul National University, Seoul, Korea; 5 Department of Molecular Medicine and Biopharmaceutical Sciences, Graduate School of Convergence Science and Technology and College of Medicine or College of Pharmacy, Seoul National University, Seoul, Korea; 6 Department of Psychology, Kangwon National University, Chuncheon, 200-701, Korea; 7 Data Science for Knowledge Creation Research Center, Seoul National University, Seoul, Korea; 8 Department of Neurosurgery, Seoul National University College of Medicine, Seoul, Korea; 9 Donders Institute for Brain, Cognition and Behaviour, Radboud University Nijmegen, The Netherlands

## Abstract

Neuronal oscillations provide a window for understanding the brain dynamics that organize the flow of information from sensory to memory areas. While it has been suggested that gamma power reflects feedforward processing and alpha oscillations feedback control, it remains unknown how these oscillations dynamically interact. Magnetoencephalography (MEG) data was acquired from healthy subjects who were cued to either remember or not remember presented pictures. Our analysis revealed that in anticipation of a picture to be remembered, alpha power decreased while the cross-frequency coupling between gamma power and alpha phase increased. A measure of directionality between alpha phase and gamma power predicted individual ability to encode memory: stronger control of alpha phase over gamma power was associated with better memory. These findings demonstrate that encoding of visual information is reflected by a state determined by the interaction between alpha and gamma activity.

Which dynamical mechanisms serve to gate information in the brain? It has recently been demonstrated that gamma activity (30–100 Hz) reflects feedforward processing, whereas slower oscillations in the alpha and beta bands reflect feedback control^1^,^2^,^3^,^4^,^5^. However, it remains unknown how these oscillations interact. Here we ask if cross-frequency coupling (CFC) between the phase of the alpha oscillations and the power of the gamma activity is involved in encoding of information from the visual to the memory system.

The CFC between low- and high-frequency oscillations has been proposed to coordinate neural processing, such that excitability of neuronal processing reflected by gamma activity is phase-locked to theta or alpha oscillations^3^,^6^,^7^. Coupling between gamma activity and the phase of theta or alpha oscillations has been reported in several MEG studies^8^,^9^,^10^, and in invasive recordings from humans and non-human primates^4^,^11^,^12^,^13^,^14^,^15^,^16^,^17^,^18^,^19^. The CFC has been proposed to play a fundamental role for organizing neuronal processing in space and time^3^,^6^,^20^,^21^. Based on previous findings, alpha oscillations - controlled by feedback mechanisms - have been demonstrated to play an important role for shaping the functional architecture of the working brain^22^,^23^,^24^,^25^,^26^. The alpha oscillations are thought to modulate neuronal excitability in a feedback manner where a decrease in power reflects the engagement of task-relevant brain regions while an increase reflects the disengagement of task-irrelevant regions. On the other hand, gamma activity is known to reflect neuronal processing associated with perception, attention and memory^27^,^28^. While gamma activity has been proposed to reflect feedforward processing^1^,^2^,^29^, there are only a few reports on pre-stimulus effects in the gamma band. For instance, an EEG study showed that 20–45 Hz gamma power in the pre-stimulus interval predicted perception^30^. A study on non-human primates demonstrated that spike-field coherence in the gamma band of putative interneurons in V4 increased with spatial attention prior to sensory input^31^. In a recent working-memory study, it was shown that gamma power was modulated by alpha phase prior to the presentation of an anticipated visual distractor^10^. In sum, these findings suggest that alpha oscillations and their interaction with the gamma activity sets the state in visual areas which then reflects the subsequent integration of feedforward and feedback information^3^. We hypothesized that top-down modulations of posterior alpha activity supports memory formation since it controls the gating of sensory information. This feedforward gating would be reflected in the gamma band. Here we test this notion by investigating if cross-frequency coupling supports the neural dynamics associated with encoding of visual information in the memory system.

We analyzed MEG data obtained from healthy subjects performing a memory paradigm in which subjects has to encode or ignore visual stimuli according to a cue (Remember or No-Remember). These data have already revealed that an increase in sensory gating is associated with a pre-stimulus decrease in alpha power^32^. By employing a new method for cross-frequency coupling (CFC) and cross-frequency directionality (CFD) analyses^33^, we investigated if gamma power is phase-locked to alpha oscillation in early visual cortex. Further we investigated the directional interaction between alpha phase and gamma power as assessed by the CFD measure, and if this interaction would be predictive of memory performance.

## Results

**Figure 1:**
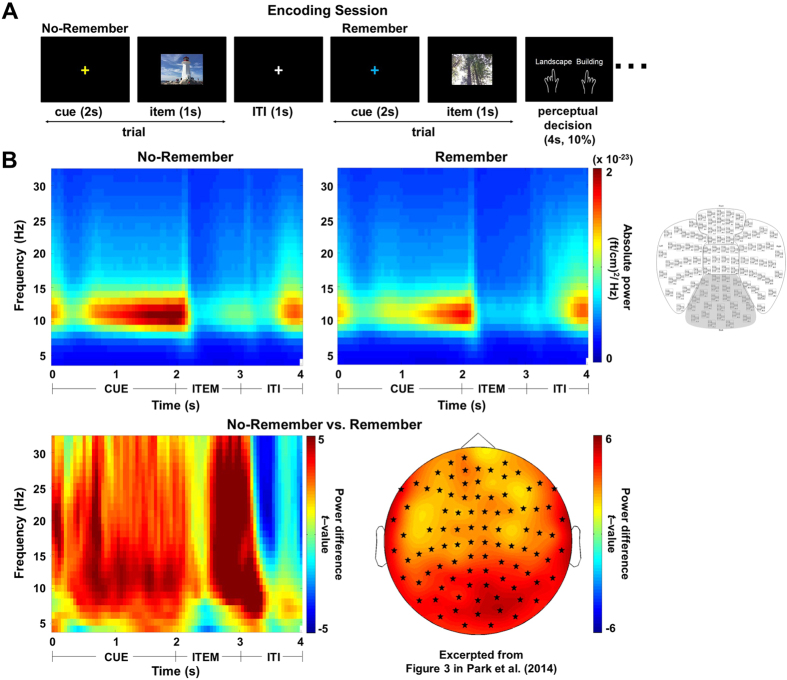
Experimental task and time-frequency analysis of power. (**A**) The cued long-term memory task. Only the encoding session of the main experimental block is depicted here (see^32^ for recognition session). Prior to each item presentation (pictures of landscapes or buildings), a Remember or No-Remember cue was presented (2 s), as indicated by the color of the fixation cross (either yellow or blue). To ensure that the subjects attended the items, perceptual decision trials were randomly presented (10% of total number of trials and they were not included in the analysis). 220 trials for each condition (Remember, No-Remember) were presented (440 trials in total). Cue and item periods in the encoding session were analyzed. For the CFC/CFD analysis, the cue period, 1 s prior to item presentation was analyzed. (**B**) Time-frequency representations of power averaged over 24 posterior sensors (defined by Neuromag layout shown in the insert on the right). The alpha power was stronger for the No-Remember compared to the Remember condition in the cue period (excerpted from Fig. 3 in^32^). The difference in alpha power was statistically significant with a posterior distribution (bottom right; 1–2 s interval; *P* < 0.05). In short, alpha power was stronger for anticipation of items to be ignored as compared to items to be remembered.

A group of healthy subjects were presented with pictures while the ongoing brain activity was recorded using MEG. Two seconds prior to each item onset, a cue indicated whether the picture should be remembered or not (Fig. 1A). Previously, we found that alpha power was strong for the No-Remember cue and relatively weak for the Remember cue (Fig. 1B). The decrease in alpha power by the cue predicted subsequent memory^32^. Note that during the item presentation there was also a strong difference in the alpha band extending to higher frequencies. Given that this effect might partly be explained by differences in the evoked response, we focused the analysis on the cue period. Here we subjected these data to a cross-frequency analysis.

### Stronger coupling between alpha phase and gamma power for Remember compared to No-Remember cues

**Figure 2:**
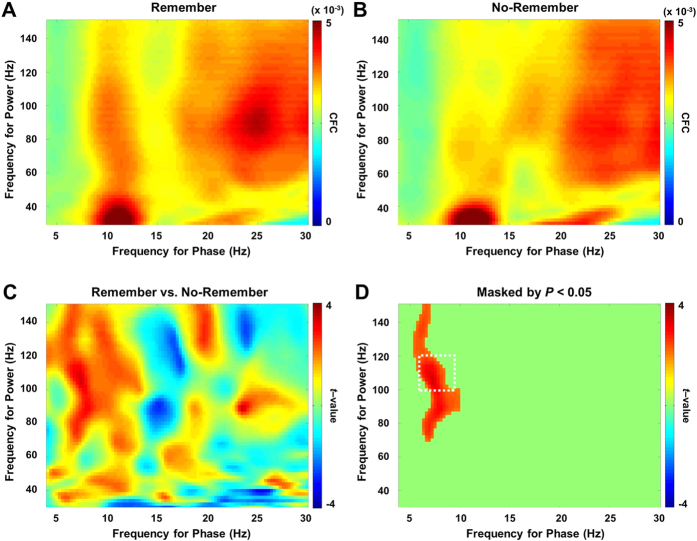
Cross-frequency coupling (CFC) during the cue period in posterior sensors. The CFC was calculated for the frequencies of interest averaged over 24 posterior sensors (the same sensors as in Fig. 1B). (**A**,**B**) The CFC for the Remember and No-Remember conditions. Phase-to-power coupling was observed in the alpha to gamma band and the beta to gamma band. (**C**) A direct comparison revealed that stronger alpha-gamma coupling was observed for the Remember compared to the No-Remember condition. (**D**) The difference was statistically significant (*P* < 0.05; controlled for multiple comparisons over frequency tiles). Importantly the CFC was strongest for the Remember condition in which the alpha power was the lowest (Fig. 1B).

To investigate the cross-frequency coupling (CFC) associated with memory encoding, we first combined all experimental conditions (Remember and No-Remember) and considered the couplings over a wide range of frequencies (phase: 4–30 Hz, power: 30–150 Hz) in 24 sensors defined by the Neuromag layout as posterior sensors (see an insert in Fig. 1B). We analyzed the 1 s cue period just prior to the picture presentation (1–2 s) and the number of trials in the conditions to be compared (Remember and No-Remember) was matched (>100 trials) for each individual. We made two observations: alpha phase at 10–12 Hz was coupled to gamma power in the 60–110 Hz range. Furthermore, the 20–30 Hz beta phase was also coupled to 60–110 Hz gamma power and 10–12 Hz alpha phase was coupled to 30–40 Hz gamma power. However, we focused the subsequent analysis on the frequency ranges displaying difference between the two conditions. The alpha-gamma coupling was stronger for the Remember (Fig. 2A) than the No-Remember (Fig. 2B) condition, which was also evident when comparing the conditions statistically (Fig. 2C). A statistical test revealed that only coupling between alpha phase and gamma power remained significant when comparing the Remember and No-Remember conditions (Fig. 2D; paired non-parametric permutation test, *P* < 0.05, controlling for multiple comparisons over frequency-by-frequency tiles). Importantly, given that alpha power was relatively low for the Remember compared to the No-Remember condition (Fig. 1B), the stronger coupling observed in anticipation of memory encoding cannot be explained by a higher signal-to-noise ratio in the alpha band. This is also important in the context of concerns raised by a recent paper^34^, which argues that non-sinusoidal oscillations can create spurious cross-frequency coupling. The observed increase in cross-frequency coupling with a decrease in alpha power argues against this concern. Our findings suggest that a decrease in alpha power is associated with stronger coupling between alpha phase and gamma power that in turn may support a gating mechanism.

**Figure 3:**
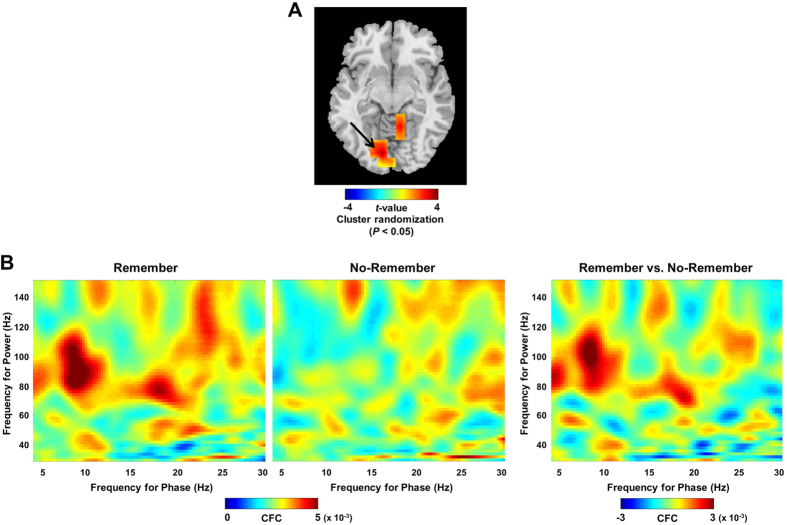
Source analysis of the CFC between alpha phase and gamma power. (**A**) To identify the sources of the CFC we used a LCMV beamformer applied to every grid point of the discretized brain volume. For the time-courses of the beamformer output we then calculated the CFC. The CFC mapped onto a standard brain MRI for the 7–10 Hz by 100–120 Hz range (white boxes in Fig. 2D). This revealed a source in early visual cortex with local maximum t-value in the left lingual gyrus (BA 18, MNI coordinates = [−10 −80 −10], Talairach coordinates = [−10 −78 −5]; paired non-parametric permutation test, *t* = 3.89, *P* < 0.05, corrected for multiple comparisons over grid points). (**B**) The CFC analysis applied to the signals in the left lingual gyrus for the conditions Remember, No-Remember, and the comparison (Remember versus No-Remember).

To identify the brain areas producing cross-frequency coupling, we used a LCMV beamformer approach. This method allows the construction of spatial filters for an individual brain volume discretized in a grid. For each grid point we calculated the CFC (phase: 4–30 Hz, power: 30–150 Hz). Based on the sensor level results, we selected a 7–10 Hz (phase) by 100–120 Hz (power) range (a white box in Fig. 2D) for which we mapped the CFC measure back to the individual brain structures. These were then morphed to a standard brain, averaged, and subjected to a statistical analysis. The most significant effect was observed in the left lingual gyrus when comparing the CFC for the Remember versus No-Remember condition (Fig. 3A; BA 18, MNI coordinates = [−10 −80 −10]; Talairach coordinates = [−10 −78 −5]; paired non-parametric cluster permutation test, *t* = 3.89, *P* < 0.05, controlling for multiple comparisons over grid points). Next, we applied the spatial filter to extract the time-course of the neuronal data from the grid point with the maximum CFC in the lingual gyrus. There was also an effect in the right cerebellum. While the involvement of the cerebellum is consistent with previous reports (see^35^), we chose to focus on the lingual gyrus given the uncertainties associated with source modelling^36^. As shown in Fig. 3B, the signals from the lingual gyrus demonstrated strong alpha-gamma coupling for the Remember condition that was absent for the No-Remember condition. The significance of the difference between the Remember and No-Remember conditions in the selected frequency tile (7–10 Hz by 100–120 Hz) was statistically assessed (paired *t*-test, *t* = 3.85, *P* = 0.001). Note that this statistical difference was expected since we selected the grid points with the strongest difference (for a rigorous statistical assessment please refer to Fig. 2D). One might ask at which alpha phase the gamma power is strongest. This is however difficult to answer since absolute alpha phase is not well-defined as it is dependent on the orientation of the dipole used in the beamformer analysis. Likewise, at the sensor level data the alpha phase is dependent on which side of the dipole the phase is estimated. We conclude that the lingual gyrus is a critical region for modulating the encoding of visual information by alpha-gamma coupling.

### Directional coupling between alpha phase and gamma power predicts subsequent memory

Next we asked if the phase of the alpha oscillations was driving the gamma power, or vice versa. The first possibility would suggest that it is the release of inhibition within an alpha cycle that determines the onset of the gamma activity. The second possibility would suggest that bursts of gamma power phase-adjust the ongoing alpha oscillations. We quantified this by using a measure of cross-frequency directionality (CFD), which is a measure based on the phase-slope index calculated between the phase of slower oscillations and the power envelope of faster oscillations^33^. Positive CFD values indicate that the phase of the slower frequency drives the power of the faster frequency, whereas negative values indicate the reverse (see the Methods for details).

**Figure 4:**
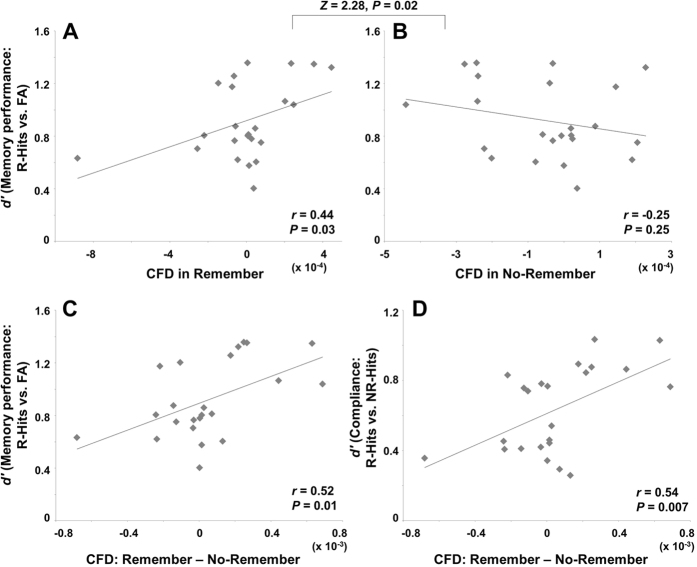
Behavioral correlates of the measure of CFD (cross-frequency directionality) for the signals from left lingual gyrus. We used the CFD measure to access whether the phase of alpha oscillations was driving the gamma power or vice versa. (**A**) Correlation between memory performance (*d*-prime) and CFD for the Remember condition across individuals. This demonstrated a significant positive correlation indicating that individuals with a positive CFD, i.e., alpha phase drives gamma power in the Remember condition, have better memory (Pearson’s correlation; *r* = 0.44, *P* = 0.03). When removing an outlier (data point for CFD −8.82^−4^), this correlation remained significant (Pearson’s correlation; *r* = 0.43, *P* = 0.04). (**B**) The correlation between memory performance (*d*-prime) and CFD for the No-Remember condition was not significant (Pearson’s correlation; *r* = −0.25, *P* = 0.25). To compare the correlations between Remember and No-Remember, we performed a *Z*-test between the *r*-values. This demonstrated that the correlations between the two conditions are significantly different (*Z* = 2.28, *P* = 0.02). This implies that better memory performance is supported by alpha phase driving gamma power for the Remember condition, whereas gamma power drives alpha phase more so for the No-Remember condition. (**C, D**) To test if these relationships were specific to the Remember condition, we correlated memory performance with the difference of CFD for the Remember and No-Remember conditions (**C**). This demonstrated a positive correlation (Pearson’s correlation; *r* = 0.52, *P* = 0.01). In addition, we correlated this difference with the ‘compliance measure’ (**D**). Compliance is assessed by using the *d*-prime calculated on the basis of later hits for the Remember cue (R-Hits) versus that for later hits for the No-Remember cue (NR-Hits); this measure quantifies how well subject comply with the memory cue. This analysis also showed a significant correlation over subjects (Pearson’s correlation; *r* = 0.54, *P* = 0.007). These findings demonstrate that when gamma power drives alpha phase, this has negative consequences for memory formation; however, when alpha phase is in control of gamma, this facilitates memory.

Is the directionality measure predictive of memory performance? To explore this, we first calculated the CFD for signals from the maximum coordinate in the lingual gyrus (MNI coordinate = [−10 −80 −10]; obtained using the LCMV spatial filter). The spatial filter was applied in order to improve the signal-to-noise ratio and isolate the contribution from the lingual gyrus. The CFD measure at the sensor level data did provide similar result but they were less robust. We then correlated the CFD for each condition with the measures of memory performance. We first considered *d*-prime in relation to CFD for the Remember condition. We found a significant positive correlation between CFD (7–10 Hz by 100–120 Hz) and *d*-prime for the Remember condition (Fig. 4A; Pearson’s correlation; *r* = 0.44, *P* = 0.03), i.e., participants in which alpha phase drives gamma power (a positive CFD) in the Remember condition show better memory (when we removed the outlier for CFD  ~ −8.82^−4^ the correlation remained significant; Pearson’s correlation; *r* = 0.43, *P* = 0.04). The mean of the CFD across the participants for the Remember condition was positive (3.91^−5^ ± 3.65^−5^ when the outlier is removed) suggesting that alpha phase is in control of gamma power. This suggests that it is the release of inhibition within an alpha cycle that controls the timing of the gamma activity. However, we did not find any relationship between CFD and *d*-prime in the No-Remember condition (Fig. 4B; Pearson’s correlation, *r* = −0.25, *P* = 0.25), where the CFD tended instead to be negative (−4.98^−5^ ± 3.59^−5^). We then performed a *Z*-test between the correlation coefficients (*r*-values) for the Remember and No-Remember conditions, which resulted in a significant difference (*Z* = 2.28, *P* = 0.02). This implies that if alpha phase controls gamma power (as in the Remember condition) the cortex enters a state of improved memory performance. The difference in CFD between the Remember and No-Remember conditions was associated with better memory performance for the Remember condition (Fig. 4C; Pearson’s correlation; *r* = 0.52, *P* = 0.01). In addition, we found that the difference in CFD between Remember and No-Remember conditions was correlated with individual differences in memory compliance (Fig. 4D; Pearson’s correlation; *r* = 0.54, *P* = 0.007), as assessed by calculating the *d*-prime on the basis of later hits for the Remember cue (R-Hits) versus later hits for the No-Remember cue (NR-Hits). This measure quantifies how well subjects selectively encoded in compliance with the memory cue^32^.

In sum, Fig. 4C,D suggest that individuals with bigger directional differences between the Remember and No-Remember cue performed better in the memory task as instructed by the cues. Considering that these effects are observed during the cue period, we conclude that anticipatory modulations in directional interactions between alpha phase and gamma power have consequences for memory encoding. In particular, when gamma power drives alpha phase, this has negative effects on selective memory formation; however, when alpha phase is in control of gamma power, this facilitates memory.

We also performed a similar analysis for the interval after the onset of item presentation; however, the effects were consistent but not as robust as during the cue period (Supplementary Fig. 1). Thus, the CFC during the cue period seems to have stronger consequence for selective memory encoding than during the item interval. Also with respect to the CFD, one might have expected that during the item interval the gamma power elicited by the stimulus would adjust the phase of the alpha oscillations. However, we were not able to identify a robust CFD during the item interval.

In addition, one might argue that the CFC is possibly induced by spectral components of event-related fields (ERF). To reduce this concern, we performed a time-frequency power analysis of the ERFs (Supplementary Figs 2 and 3). These analyses verified that the CFC effects are not explained by the oscillatory components of evoked responses elicited by the picture stimuli.

## Discussion

Here we demonstrate that gamma power is coupled to the phase of alpha oscillations in early visual areas (lingual gyrus; BA 18) in anticipation of visual items in a memory paradigm. Importantly, the coupling increased in anticipation of memory items to be encoded, as compared to items to be ignored. This increase in coupling was associated with a decrease in alpha power. Furthermore, better memory performance was predicted by alpha phase driving gamma power during the anticipation of upcoming memory items.

### Alpha-gamma coupling sets up a cortical state facilitating subsequent memory encoding

Recently, it has been reported that gamma oscillations reflect a feedforward drive in the visual hierarchy, whereas slower oscillations in the alpha and beta bands reflect feedback processing^1^,^2^,^3^. These findings are based on intracranial recordings in monkeys in which directional measures have been applied^1^,^2^. Further, it is consistent with human EEG and MEG findings. Spatial cues can modulate the alpha band activity therefore reflecting top-down driven feedback control^37^. Gamma activity becomes particularly strong in response to visual stimuli suggesting a feedforward drive^38^. However, the dynamics reflecting the integration between feedforward and feedback signals remain unknown. Our findings suggest that gamma power phase-locked to alpha oscillations prepares sensory regions for this integration. In particular, the alpha-gamma coupling sets up a cortical state that facilitates a subsequent picture to be retained. In this case, feedback control is clearly reflected in the alpha band as was shown here to be modulated by the cue. To the best of our knowledge, this study is the first to report on task-modulated coupling between alpha phase and gamma power in the context of long-term memory encoding as well as the directional interactions between these two rhythms.

### Cross-frequency directionality predicts subsequent memory performance

We observed that better subsequent memory performance was predicted by cross-frequency directionality in terms of alpha phase driving gamma power during the anticipation of to-be-remembered items for the Remember condition (Fig. 4A). Even though the inverse relationship between these physiological observations and memory performance was not significant for the No-Remember condition, the correlation coefficient between the two conditions (Remember versus No-Remember) was significantly different. Furthermore, the differential directionality measure between the two conditions was significant not only for selective encoding performance (Fig. 4C), but also for compliance with the cue instruction (Fig. 4D). This suggests that in good memory performers, alpha takes control over gamma in order to support selective memory encoding.

### Cross-frequency coupling as general finger-print of neuronal processing

The coupling between phase of slow oscillations (theta, alpha band) and power in the gamma band has been observed in various regions during rest^8^,^9^,^12^,^14^, processing of visual^13^,^39^ and auditory stimuli^4^, memory operations^7^,^15^,^16^,^40^, working memory maintenance^35^, and even *in vitro*^41^,^42^. These findings suggest that the interaction between slow and fast oscillations play an important role in coordinating information processing.

### Cross-frequency interactions reflect top-down control

Converging evidence suggests that alpha activity serves to gate input in a feedback-controlled manner. For example, an intriguing study on visual working memory using TMS and EEG has shown that the phase dynamics of posterior alpha oscillations prior to stimulus encoding is predictive of behavioral performance^43^. This finding relates to the present study, where the coupling between alpha phase and gamma power is stronger during the pre-stimulus (cue) period than during item presentation. In particular, our finding that alpha phase driving gamma power predicts better memory in anticipation of the visual items supports this notion.

### Concerns about artifactual cross-frequency coupling

It has previously been argued that measures of phase-to-power couplings are sensitive to ‘non-sinusoidal’ oscillations^34^,^44^. In particular, harmonics of the phase-providing oscillations (alpha oscillations in this case) can produce a spurious coupling which can wrongly be interpreted as a phasic modulation of physiological gamma activity. Such concerns can be ruled out in our study. First, the alpha phase to gamma power coupling was strongest in the condition when alpha power was lowest. This reduces the concern that the coupling stems from non-sinusoidal oscillations, since the artifacts would be more pronounced when alpha power is stronger. Second, the coupling and directionality results were obtained via a comparison between experimental conditions, where the number of trials was matched (>100 trials) for each individual. As such, our results are not subject to biases resulting from different trial numbers between conditions. Third, we report on cross-frequency coupling during the pre-stimulus cue period (see Supplementary Fig. 1 for CFC results during item interval). Therefore the coupling cannot be explained by spectral components of the evoked response due to the presentation of visual stimuli.

In conclusion, we have identified robust cross-frequency coupling between alpha phase and gamma power that is predictive of selective memory encoding. The cross-frequency coupling reflects a physiological mechanism setting the state which controls the subsequent encoding of visual input to memory. In particular, a cue-driven suppression of alpha power resulted in increased alpha-gamma coupling, which supported memory encoding.

## Methods

### Participants

The data were obtained from 23 participants (12 females; mean age of 24.8 ± 3.1 years). Analysis of these data were also presented in a previous report^32^. None of the participants had a history of developmental, psychological, or neurological disorders. They all had normal or corrected-to-normal vision and were right-handed. All participants provided written informed consent and received monetary compensation for their participation. The present study was approved by the Institutional Review Board (IRB) at Seoul National University Hospital (IRB No. C-1007-156-325) and conducted in accordance with the ethical guidelines that have their origin in the Declaration of Helsinki.

### Stimuli

Six hundred and forty real-life photographs of landscapes and buildings^45^ were used as stimuli; these did not include well-known landscapes and buildings. Pictures with a visual angle of 8° horizontally (334 × 250 pixels) were projected to a screen using STIM2^TM^ software (Compumedics Neuroscan, Charlotte, NC). The stimuli were evenly divided into three sets to be used for the conditions: Remember, No-Remember and New. The Remember and No-Remember conditions were used in both the encoding and recognition sessions and the New condition was used only in the recognition session. The stimuli belonging to the three sets were counterbalanced over subjects.

### Experimental paradigm

The experiment consists of two experimental blocks - a training block followed by an experimental block. As in standard memory paradigms, each study block consisted of encoding, interference, and recognition sessions.

In the encoding session (Fig. 1A), 440 pictures (220 pictures for each Remember and No-Remember conditions) were presented. A cue was shown for 2 s in which the color of the fixation cross indicated either to remember (e.g. blue) or not to remember (e.g. yellow) the upcoming picture. The color of the cue was counterbalanced across subjects. Then a picture was presented for 1 s followed by a 1-s inter-trial interval (ITI). In 10% of the trials independent of the cue, we asked the subjects to make a perceptual decision on whether the picture was a landscape or a building. This was to ensure that the subjects perceived the presented stimuli. To prevent motor preparation confounds, they were instructed to press the left or right button (left or right index finger) as instructed by the question screen. This question screen was turned off as soon as a response was provided, for a maximum display of 4 s. The perceptual decision trials were not used in the MEG analysis. To reduce recency effects, a short interference task (arithmetic calculation) followed which lasted 5 minutes.

In the recognition session, the 440 stimuli from the encoding session were randomly intermixed with 200 new stimuli. Each picture was presented for 4 s and the subjects were instructed to indicate if the picture was presented before (old) or not (new). The fixation screen then followed (1 s). The subjects used the index, middle, and ring fingers of the right hand, which were already associated with the appropriate response (i.e., old, don’t know, and new) during the training block. The fingers associated with the three responses were counterbalanced across subjects. To reduce guesses, they were instructed to press the “don’t know” button when they were uncertain. Subjects were instructed to respond old or new, independently of the cue; thus they were supposed to respond old, even if they remembered that the item with a No-Remember cue. The picture disappeared as soon as a response was made with a maximum display for 4 s. The total duration of the experiment was approximately 100 minutes.

In the training block, 160 pictures were used, and the stimuli from No-Remember were not tested in the recognition session. The subjects were therefore naïve about the later test on No-Remember items in the recognition session of the main experimental block.

### Behavioral measurements

We computed two behavioral measures; d-prime and compliance (see^32^). The *d*-prime was calculated for later Hits for the Remember cue (R-Hits) versus False Alarm (FA) rates; Z (R-Hits) – Z (FA); Z refers to the inverse of the cumulative distribution function. Compliance was assessed by using the *d*-prime measure calculated on the basis of later Hits for the Remember cue (R-Hits) versus later Hits for the No-Remember cue (NR-Hits); Z (R-Hits) – Z (NR-Hits). This measure quantifies how well subjects comply with the memory cue.

### MEG data recording

Data recordings were acquired with a whole-head MEG Neuromag (VectorView^TM^, Elekta Neuromag Oy, Helsinki, Finland) at a 1200 Hz sampling rate. The vertical and horizontal electrooculogram (EOG) and electrocardiogram (ECG) were recorded for subsequent exclusion of eye and cardiac artifacts. Head position indicator (HPI) coils were attached to the head of each subject, and anatomical landmarks (nasion and bilateral preauricular points) were spatially identified by a 3D digitizer (FASTRAK^TM^, Polhemus, Colchester, VT). Then the subject’s head position was registered by localizing HPI coils in the MEG device for later source reconstruction with high precision. Before data analysis, a Maxwell filter (Signal Space Separation), which separates brain-related and external inference signals, was applied to reduce the confounding influence of biological and environmental noises^46^,^47^.

### Data analysis

The data analysis was performed in Matlab 2013b (MathWorks, Natick, MA) using the Fieldtrip toolbox^48^ (http://fieldtrip.fcdonders.nl), and in-house scripts according to recently published guidelines^49^. Before the analysis, the data were downsampled at 600 Hz after applying a low-pass filter at 200 Hz. Trials contaminated by ocular artifacts, SQUID jump, and muscle artifacts were manually rejected using visual inspection. Additionally, remaining electrooculographic (EOG) and electrocardiographic (ECG) artifacts were reduced using independent component analysis (ICA).

### Source analysis

Individual head models were created from anatomical MRIs using segmentation in Fieldtrip and SPM8 (http://www.fil.ion.ucl.ac.uk/spm). Realistically shaped single-shell descriptions of the brains were constructed from each individual’s MRI^50^. The brain volume of each individual subject was divided into a grid with 0.8 cm resolution, and normalized to the template MNI brain (International Consortium for Brain Mapping, Montreal Neurological Institute, Canada). We used a LCMV (linearly constrained minimum variance) beamformer^51^ to create spatial filters for extracting the time course from a given region. The lead field was calculated for each grid point. A common filter was used for the different conditions. Prior to calculating the filter, the mean of the data was removed, but they were not filtered otherwise. A regularization of lambda 7% was applied.

### Cross-frequency coupling (CFC) and directionality (CFD) analysis

The measure of CFC is based on coherence between the phase of low-frequency and power of high-frequency signals^8^. We have recently developed a new method for assessing if the phase of the slower oscillations controls the power of the faster activity, or vice versa. This method is termed cross-frequency directionality (CFD)^33^ and was applied in this study. The CFD measure is based on phase-slope index (PSI)^52^, which was initially proposed for assessing the direction of coupling between two oscillatory signals of similar frequencies. For instance, assuming that an oscillator operating in a frequency range *f*_*a*_ to *f*_*b*_ (e.g. 8–13 Hz) drives another oscillating system with a fixed time lag, there is a phase difference between the two systems. Importantly, the phase difference changes linearly with frequency in the range *f*_*a*_ to *f*_*b*_^53^,^54^. PSI estimates the slope of phase difference as a function of frequency in a given frequency band. The sign of the slope reflects the directional interaction. A positive slope of the phase difference means the first oscillator drives the other, whereas negative means the reverse.

To produce reliable task-specific CFC and CFD results, we first matched the number of trials in the conditions to be compared (Remember and No-Remember) for each individual. The average number of trials across subjects was 151.65 ± 19.21 (range 116 to 181). This is a large enough number of trials to get robust effects, and we applied the analysis during both cue and item periods, each being 1 s long.

### Estimation of time course of power at frequency *v*

The temporal evolution of power at frequency *v* is termed 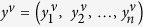. For both CFC and CFD, *y*^*v*^ is compared to the phase of the signal *x*. As such, *v* constitutes frequencies on the y-axis in a CFC diagram. This set of frequencies was initially chosen to range from 10 to 150 Hz in 2 Hz increments: *v* = (10, 12, 14,…, 150). The power as a function of time is estimated by applying a discrete Fourier transform to successive segments of the data with *M* samples (sliding time window). Prior to the Fourier transform, each data segment is multiplied by a Hanning taper *h* to reduce spectral leakage:


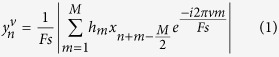


*F*_*s*_ is the sampling frequency. The length of *M* is typically chosen to be *w *=* *5 cycles long with respect to the frequency *v*, i.e. 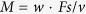.

### Quantifying cross-frequency coupling (CFC) and cross-frequency directionality (CFD)

Let ***x*** and ***y***^*v*^ denote the fast Fourier transform (FFT) of signal *x* and *y*^*v*^ respectively. The length of FFT is *n*_*FFT*_ and the frequency resolution Δ*f* is 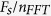. Let 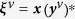 be defined as the cross spectrum between ***x*** and ***y***^*v*^ where ‘*’ denotes the complex conjugate. These Fourier transform vectors are centered at frequencies 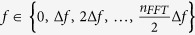.***x***(*f*), ***y***^*v*^(*f*) and 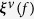 represent the vector elements centered at frequency *f*. The CFC can be quantified by estimating the coherence between the signal *x* centered at *f* and power envelope of the signal for frequency *v* in the following way:


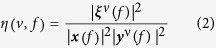


As we described above, the estimation of the CFD is based on the phase-slope index (PSI)^33^. We applied the PSI to the signal *x* and the power envelope of the signal *y*^*v*^at frequency tile(*v*, *f*):


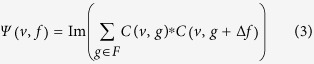


with
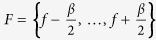
.
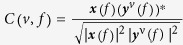
 is the complex coherency and Im denotes the imaginary part. We used a *β* of 2 Hz. These parameters were found appropriate in simulation studies and when applied to ECoG data^33^. Increasing *β* of 4 Hz yielded comparable results.

### Group statistics

Group statistical analysis was performed on the CFC and CFD data of 23 participants using non-parametric randomization statistics in Fieldtrip (Monte Carlo randomization)^55^, which effectively controls for the Type I-error rate with respect to multiple comparisons over the frequency combinations. The CFC and CFD measures were not baseline-corrected when conditions were compared. Individual data were subjected to dependent-samples *t*-test (Remember versus No-Remember) for CFC, and to one sample *t*-test against zero for each condition for CFD analysis.

For the first-level statistics, sensors or voxels below a threshold (*t*-test; *P* < 0.05) were identified from the *t*-statistics. Subsequently spatially contiguous sensors or voxels below this threshold were defined as a cluster. Then, the sum of the *t*-values for a given cluster was used for the cluster-level statistics.

For the first-level statistics, sensors or voxels below a threshold (*t*-test; *P* < 0.05) were identified from the *t*-statistics. Subsequently spatially contiguous sensors or voxels below this threshold were defined as a cluster. Then, the sum of the *t*-values for a given cluster was used for the cluster-level statistics. The null distribution was estimated using 500 randomizations, and correction for multiple comparisons was performed at the cluster level (*P* < 0.05).

## Additional Information

**How to cite this article**: Park, H. *et al*. Formation of visual memories controlled by gamma power phase-locked to alpha oscillations. *Sci. Rep.* **6**, 28092; doi: 10.1038/srep28092 (2016).

## Supplementary Material

Supplementary Information
