# Supplementary material for: Formation of visual memories controlled by gamma power phase-locked to alpha oscillations
Source: Sci Rep. 2016 Jun 16;6:28092. doi: 10.1038/srep28092 (PMC4910116; doi:10.1038/srep28092)
Supplement: Supplementary Information [file srep28092-s1.pdf]

Supplementary Information For:

Formation of visual memories controlled by gamma power phase-locked to alpha oscillations

Hyojin Park,[1,2,3,4] Dong Soo Lee,[1,3,4,5,*] Eunjoo Kang,[6] Hyejin Kang,[1,7] Jarang Hahm,[1,3,4] June Sic Kim,[8] Chun Kee Chung,[4,8,*] Haiteng Jiang,[9] Joachim Gross,[2] Ole Jensen [9,*]

[1]Department of Nuclear Medicine, Seoul National University College of Medicine, Seoul, Korea

[2]Institute of Neuroscience and Psychology, University of Glasgow, Glasgow, United Kingdom

[3]Institute of Radiation Medicine, Medical Research Center, Seoul National University, Seoul, Korea

[4]Interdisciplinary Program in Cognitive Science, Seoul National University, Seoul, Korea

[5]Department of Molecular Medicine and Biopharmaceutical Sciences, Graduate School of Convergence Science and Technology and College of Medicine or College of Pharmacy, Seoul National University, Seoul, Korea

[6]Department of Psychology, Kangwon National University, Chuncheon, 200-701, Korea

[7]Data Science for Knowledge Creation Research Center, Seoul National University, Seoul, Korea

[8]Department of Neurosurgery, Seoul National University College of Medicine, Seoul, Korea

[9]Donders Institute for Brain, Cognition and Behaviour, Radboud University Nijmegen, Nijmegen, The Netherlands

* Corresponding authors:

Dong Soo Lee: dsl@snu.ac.kr

Chun Kee Chung: chungc@snu.ac.kr

Ole Jensen: ole.jensen@donders.ru.nl

Supplementary Figure 1.

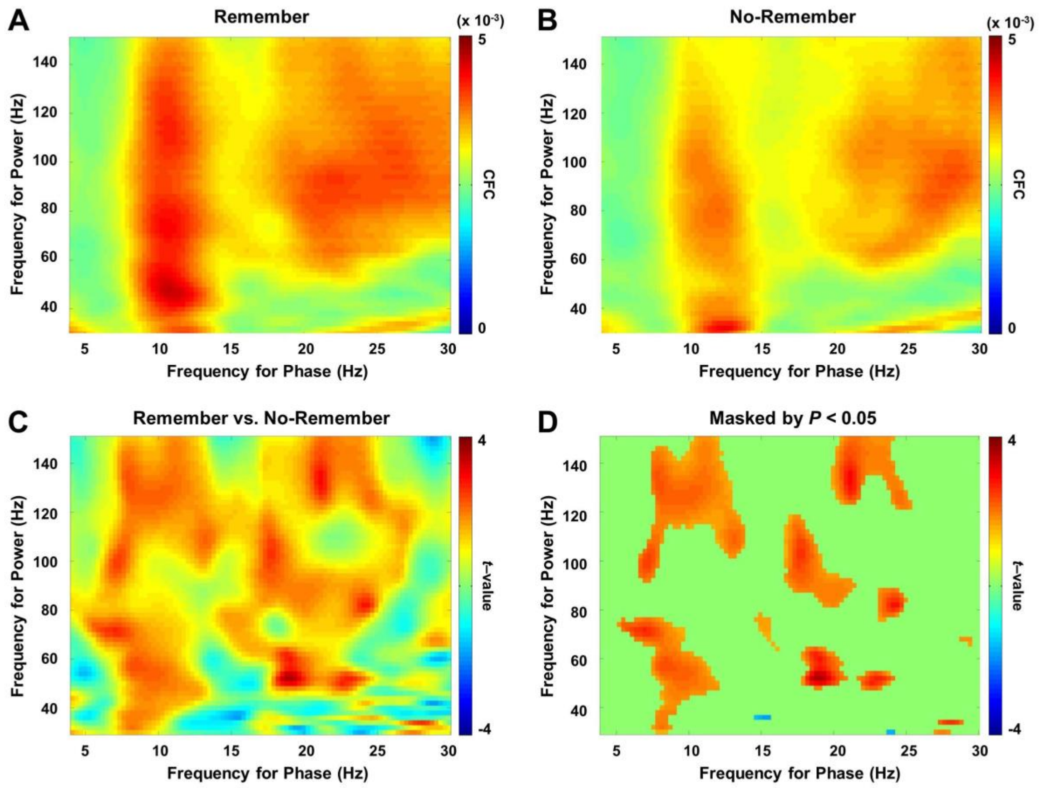

Cross-frequency coupling (CFC) during item (2-3 s) in posterior sensors. We performed the same CFC analysis during item interval across the same posterior 24 sensors. However, the results were not as robust as during cue period. **(A and B)** CFC for Remember and No-Remember. Similar to CFC during cue, we found task-specific CFC that showed increased couplings between alpha phase and gamma power, and beta phase and gamma power. This was stronger for the Remember condition. When compared to the CFC during the cue (Fig. 2B and 2C), the CFC during the item presentation was stronger for both conditions. **(C and D)** When statistically compared between the conditions, the overall coupling effects seem stronger for the Remember condition. However, unlike the cue period, statistical significance did not survive correction for multiple comparisons (paired non-parametric permutation test, $P < 0.05$, not corrected for multiple comparisons).

Supplementary Results

Time-frequency representation (TFR) of event-related fields (ERF)

To reduce the concern of spurious CFC, which might possibly be induced by spectral components of event-related fields (ERF), we performed time-frequency power analysis of ERF. If, in this analysis, spectral components of both alpha and gamma ranges are observed for the same condition (Remember or No-Remember), and during the same period (either cue or item), the CFC results for the same condition and interval would be of concern because the CFC between alpha and gamma could have been induced by the evoked components elicited by visual stimulus presentation. First, we analyzed event-related fields for each condition using the whole epoch of the trial, and then this was handed over to the time-frequency analysis. Since our main concern lies with components evoked by the memory item, the baseline used was 1.6-1.8 s (before item presentation). We performed this analysis separately for low- (4-30 Hz) and high-frequency (30-150 Hz) ranges for applying different resolution.

Supplementary Figure 2.

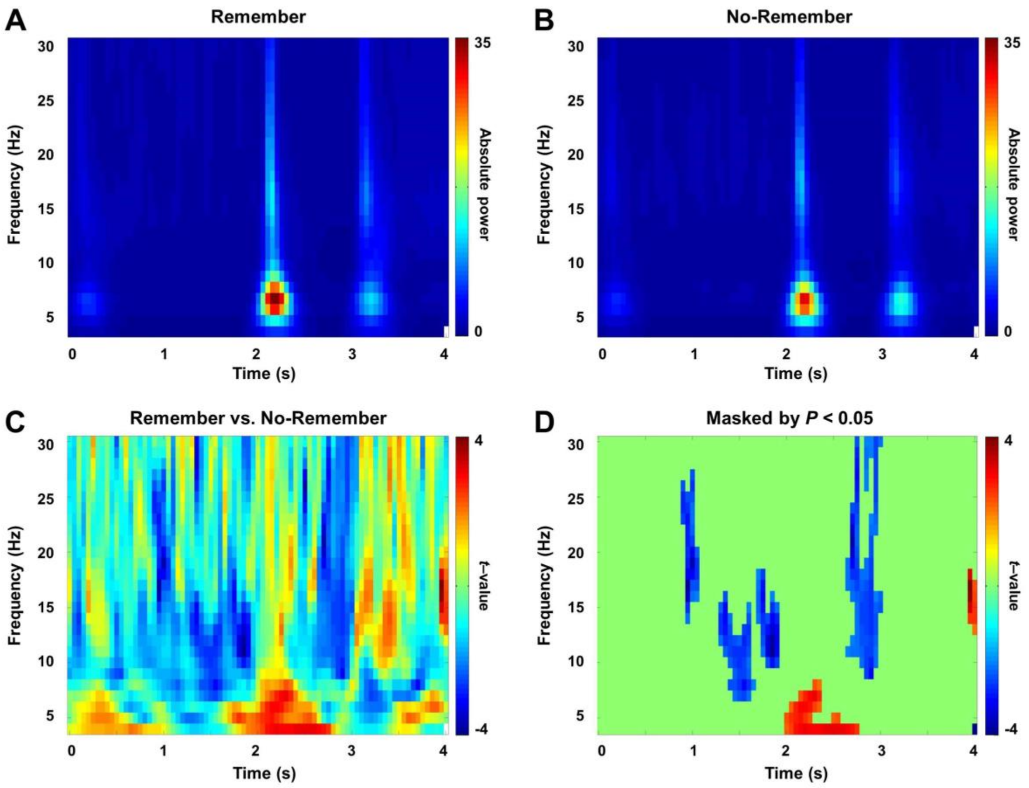

Time-frequency representation (TFR) of spectral components by evoked response for low-frequency ranges (4-30 Hz). (A and B) TFR of ERF for Remember and No-Remember conditions during whole trial duration (0-4 s). During an early period of item presentation, spectral components of alpha frequency ranges were observed for both Remember and No-Remember, with stronger power for the Remember than No-Remember condition. **(C and D)** When the two conditions are statistically compared, significance for the alpha component survived for the Remember condition during item interval (paired non-parametric permutation test, $P < 0.05$, corrected for multiple comparisons at cluster-level).

Supplementary Figure 3.

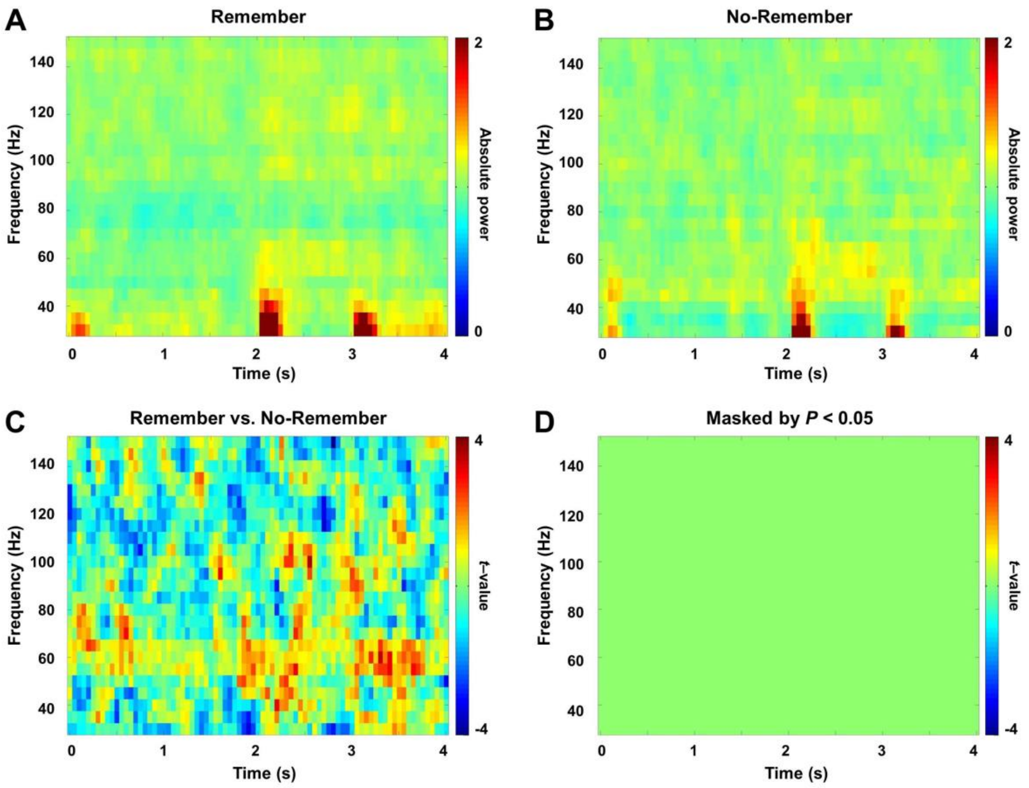

Time-frequency representation (TFR) of spectral components of evoked response for high-frequency ranges (30-150 Hz). (A and B) TFR of ERF for Remember and No-Remember conditions during the whole trial duration (0-4 s). During an early period of item presentation, spectral components of low gamma ranges (30-40 Hz) were observed for both Remember and No-Remember with stronger power for the Remember than No-Remember condition. **(C and D)** When the two conditions are statistically compared, the low gamma component was not significant (paired non-parametric permutation test, $P < 0.05$, corrected for multiple comparisons at cluster-level). In summary, we did not observe concurrent increases of alpha and gamma frequency components from the evoked responses for any condition and any interval. Thus the concern of spurious CFC is substantially reduced. Furthermore, the CFC found in this study was stronger during the cue than the item period, so our CFC results stem from anticipatory task-specific modulation.
